# Caregivers' Perspectives on Transitioning Dental Care for Adolescents with Special Healthcare Needs–A Cross-Sectional Study

**DOI:** 10.1155/2022/7296372

**Published:** 2022-08-31

**Authors:** Glenn Canares, Roxanne Clarke, Erica Caffrey, Sydnee Chavis

**Affiliations:** ^1^Clinical Assistant Professor, Division of Pediatric Dentistry, Department of Orthodontics and Pediatric Dentistry, University of Maryland School of Dentistry, 650 West Baltimore Street, Office 2217, Baltimore, MD 21201, USA; ^2^Pediatric Dentist, Division of Pediatric Dentistry, Department of Orthodontics and Pediatric Dentistry, University of Maryland School of Dentistry, 650 West Baltimore Street, Baltimore, MD 21201, USA; ^3^Clinical Assistant Professor, Division of Pediatric Dentistry, Department of Orthodontics and Pediatric Dentistry, University of Maryland School of Dentistry, 650 West Baltimore Street, Office 2212, Baltimore, MD 21201, USA; ^4^Clinical Assistant Professor, Division of Special Care and Geriatrics, Department of Oral Surgery, University of Maryland School of Dentistry, 650 West Baltimore Street, Office 1202, Baltimore, MD 21201, USA

## Abstract

**Introduction:**

Few studies have investigated the concerns of caregivers of adolescents with special health-care needs (ASHCN) regarding the barriers and challenges of transitioning from a pediatric to an adult-based dental home. The purpose of this study was to assess these perceptions.

**Methods:**

A 23-question survey was administered to guardians of ASHCN who presented to the pediatric dental clinic at the University of Maryland. Question types were either multiple choice, Likert scale responses, or open-ended. A descriptive analysis and Fisher's exact test were performed. Keywords were evaluated from the open-ended answers.

**Results:**

Twenty-seven caregivers completed the survey over a six-month period. Sixty-six percent of caregivers were aware that dental needs change as child ages and thought that transitioning was a logical next step, 78% had concerns about transitional care, and 70% did not have the desire to transition. Fisher's exact analysis comparing awareness of transition versus the desire to transition was *p* < 0.10.

**Conclusion:**

Most caregivers were aware of the changing dental needs of ASHCN and believed transitioning was a logical step. Many caregivers lacked readiness and perceived multiple barriers to transitioning. Awareness of the need to transition from pediatric to adult-based dental homes was not correlated with the readiness to transition.

## 1. Introduction

Parents and caregivers of adolescents with special health-care needs (ASHCN) typically have the responsibility of coordinating long-term dental care as their child becomes an adult. [1] This process, termed “transitional care,” is the purposeful, planned movement of adolescents, and young adults with chronic physical and medical conditions from child-centered to adult-oriented health-care systems. [2] This transition is a dynamic and multilayered process that may be complicated by factors such as provider availability, insurance coverage, and the psychosocial and behavioral characteristics of this age group. [3] Additionally, socio-economically vulnerable populations tend to have a heavier dental disease burden. [4] Such challenges may influence a parent's readiness to undergo this process, but few studies have investigated the concerns of caregivers and adolescents regarding changing dental homes [4].

Nearly 20% of U.S. children under the age of 18 years have a special healthcare need (SHCN), and one in five U.S. families have a child with an SHCN. [5] As people with SHCN become adults, their dental needs may become more complicated. Treatment plans may include periodontal therapies, advanced restorative techniques, endodontic procedures, and prosthodontic considerations for permanent teeth. [6] These treatment needs may extend beyond the scope of pediatric dentistry and require the expertise of an adult dental provider who regularly provides services for full permanent dentition [7].

Literature on the transition of ASHCN to adult-centered medical homes has addressed the perspectives of patients and caregivers, who reported low confidence in the process and difficulties in locating adult-based providers. [8–12] With regard to dental care, one study found that parents believed that pediatric dentists played an important role in the transition process. [13] Another study evaluating provider perspectives on transitioning reported that both pediatric dentists and general dentists perceive barriers to transitioning including inadequate training, limited availability of general dentists, and low reimbursement. [14, 15].

The purpose of this study was to assess the awareness and readiness to transition among caregivers of ASCHN. The null hypothesis is that there is no difference between caregivers' awareness of transition for their child and readiness to transition for their child.

## 2. Methods

This descriptive cross-sectional study was reviewed by the Institutional Review Board (IRB # HP-00084506) of the University of Maryland School of Dentistry (UMSOD), Baltimore, MD, and qualified for an exemption.

A paper survey was administered to parents and guardians of a pediatric patient at UMSOD, aged 12 years or older that has SHCN. The survey's questions were categorized according to demographics, awareness, concerns/expectations about transitioning, and readiness or desire to transition. The questionnaire consisted of 23 total questions including the age of the child, type of residence, degree of dependency of the child on the caregiver, and transition readiness. This included 11 multiple choice questions, 8 Likert scale responses ranging from “Strongly Disagree” to “Strongly Agree,” and 4 open-ended questions. No personal identifying information was recorded or collected.

The inclusion criteria for the study were as follows: a parent or guardian of a pediatric patient at UMSOD, aged 12 years or older that has SHCN. Patients with SHCN, by definition, included any adolescent with behavioral, congenital, developmental, or cognitive disorders, and systemic diseases. [16] The exclusion criteria for the study were as follows: a parent or guardian who did not consent to participate in the survey; a parent or guardian of a child who did not have SHCN; a parent or guardian of a child with SHCN who is younger than age 12; a parent or guardian of a child who is not a patient at the UMSOD in the Pediatric Dentistry clinic, or a parent or guardian was not present at the dental appointment of the child (not in the custody of the state).

The population for the convenience sample was identified in the clinic's electronic health record, Axium (Exan, British Columbia, Canada). The patient scheduling feature was used to locate patients who have a dental appointment in which the caregiver may qualify for the study. If a caregiver met the inclusion criteria, a written script was read to him or her and used to obtain verbal consent by a member of the research team. The research team was given instructions to read the written script verbatim. Instructions were provided for survey completion. Caregivers were informed that their participation is voluntary and that the child's dental treatment will not be affected by their response. Surveys were administered over a six-month period, by a member of the research team, or a pediatric dental resident at the Pediatric Dentistry clinic, during or immediately after the child's dental appointment. The responses were scored and recorded by that individual on the paper survey and were subsequently input on a password-protected Excel (Microsoft Inc, Redmond, WA) spreadsheet. The paper surveys were stored in a locked compartment and shredded after completion of the study.

A descriptive analysis of demographics and questionnaire results was performed for the convenience sample. Fisher's exact test was performed using SAS 9.4 (SAS Institute, Cary, NC) to evaluate the null hypothesis. Key words were evaluated from the open-ended answers.

## 3. Results

Twenty-seven caregivers completed the survey over a six-month period. For the patients with SHCN aged 12 years and older, 23 (85%) of the patients live at home with family, while 4 (15%) live in a residential facility or other places of dwelling. The patients were categorized into four different age groups (Table 1). The main questions from the survey were categorized by caregiver awareness, concerns, and readiness or desire to transition.

### 3.1. Awareness

A total of 27 (100%) of caregivers responded that dental care was important to them, with 17 responses (68%) and 8 responses (32%) from age groups 12–15 and 16–19 years old, respectively. Seventy percent of the caregivers were aware that dental needs change as a child gets older, with an awareness percentage of 71% and 75% in age groups 12–15 and 16–19, respectively. 74% of caregivers thought that transitioning is a logical step toward adulthood, with affirmative responses of 76% and 75% in age groups 12–15 and 16–19, respectively.

### 3.2. Concerns and Expectations about Transitioning

Closed and open-ended questions were asked on the questionnaire, and responses were recorded regarding concerns about transitioning. Twenty-one (78%) caregivers had concerns about transitioning dental care, regardless of the patient's age. Caregivers reported multiple responses for each open-ended question, and the answers were recorded based on common themes. The main concerns about transitioning were that the SHCN of the patient will not be met including that the patient will not adjust to a new environment (29%), interruption of dental care (4%), unknown eligibility of dental services (3%), and some caregivers (25%) had no main concerns. In the older age group (16+) with a sample of 10 caregivers, a total of 7 (70%) caregivers were more concerned about the patient adapting to a new environment, compared to 6 (55%) caregivers with no concerns in the 15 age group and younger.

**Figure 1:**
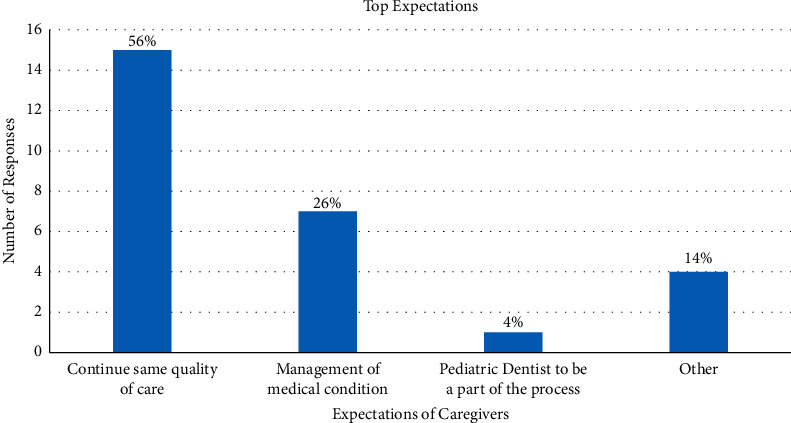
Expectations and percentages of caregivers in response to the transitioning process.

For expectations expressed by caregivers, 15 (56%) of caregivers expected that the adult-based dental setting should continue the same quality of care, 7 (26%) caregivers felt the new dental facility should be able to appropriately manage the patient's medical condition, and 1 caregiver (4%) felt that the pediatric dentist should maintain an active role in the transition process; however, 2 (7%) caregivers expected that the patient should adapt to their new environment, and 2 (7%) caregivers had no major expectations (Figure 1).

### 3.3. Readiness or Desire to Transition

Caregivers' responses of readiness or desire to transition were as follows: 17 (63%) caregivers have not thought about what will happen when the patient transitions. 19 (70%) of caregivers did not have the desire to transition now; regardless of the patient's age, however, 6 (67%) of caregivers in age group 16–19 reported no desire in transitioning though their children were old enough to start their transitioning process. In assessing caregivers' awareness of the necessity for their child to transition care versus their readiness for their child to continue care, Fisher's exact analysis demonstrated that there was not a significant difference between caregivers' awareness of transition for their child and readiness to transition for their child (*p* < 0.10) (Table 2).

## 4. Discussion

This study evaluated the perceptions of caregivers of ASHCN regarding the process of transitioning from a pediatric dental provider to an adult-based dental home. Most caregivers felt that dental care was important and was aware of transitioning as a logical next step. The assessment of caregiver readiness indicated that there was hesitation surrounding the transition process. However, per the null hypothesis, there was no difference between caregivers' awareness of transition for their child and readiness to transition for their child. Many caregivers had not yet contemplated transitioning their adolescents to adult dental providers, and most confirmed that they were not ready to do so at the time of the survey. There was no significant association between awareness of and the desire to transition. It is possible that the adolescents' degree of dependency on their caregivers may be linked with caregiver readiness to transition. A previous study showed that guardians understood the value of transitioning to comprehensive care but prioritized other caregiving responsibilities. [13] Of the caregivers whose adolescents were almost entirely dependent on them for dental needs, only a quarter of them felt ready to transition. For ASHCN with conditions such as autism, there can be a heavy dependence on the caregiver to arrange for dental care. [17] Those with less dependent adolescents were twice as likely to express readiness. These echo sentiments expressed by more typically functioning ASHCN reported in a previous study, who were more self-conscious about being treated in a pediatric setting than their peers with more extreme functional differences. [13].

There are emotional barriers to transitioning that likely contribute to the decreased readiness of caregivers surveyed in this study. One challenge is the fear by some caregivers that their adolescents would not adjust well to a new environment, a concern that is consistent with findings from previous studies. [18] Caregivers may anticipate a less friendly or welcoming dental atmosphere once their adolescent has transitioned to an adult-centered facility. [19] All caregivers surveyed stated that their dental needs were met, their adolescent was treated well, and the treatment was affordable and of good quality. They also valued provider comfort, patience, and experience, and many of them stated an expectation of the same quality of care once the patient transitioned. The bond between the pediatric dentist and the adolescent patient is a reported hindrance to transitioning from both the provider and patient perspective [3, 19].

Logistical obstacles to readiness to transition include worries about inconsistencies in dental treatment that would come with a change in provider and setting. There are reported concerns by children and parents that adult-based healthcare providers will have different treatment recommendations. [20] Furthermore, the management of adolescents with behavioral challenges or medical complications may necessitate dental treatment under sedation or general anesthesia. Access to operating room time is inadequate while hospital credentialing rules, policies for allowing hospital privileges, and limited insurance coverage for general anesthesia severely limit the number of ASCHN who can obtain needed dental treatment [21, 22].

There were limitations to this study. The group surveyed for this study was a convenience sample, and thus, the results are not generalizable beyond the dental clinic at UMSOD. Additionally, the extent of the adolescents' functional limitations was not fully assessed. Although the question of dependency on a caregiver for dental needs was asked, specific details regarding the adolescent's ability to self-manage care were not. Response and subject bias may have influenced the survey answers given.

There is currently a lack of evidence-based protocols to guide transition decisions and planning for dental providers. [1] These data on caregiver perspectives may help facilitate the creation of effective strategies to ensure a more comprehensive and successful transition for all ASHCN. Furthermore, research investigating the perceptions of caregivers of ASHCN regarding oral health care will be helpful to understand the challenges involved in transitioning to adult-centered providers. Development and expansion of transition planning among relevant stakeholders are essential to improve oral health outcomes and greater health equity for those with SHCN.

## 5. Conclusions

1. Most caregivers were aware of the changing dental needs of their child with SHCN and viewed the transition from pediatric to adult-based dental homes as the next logical step
2. Awareness of the need to transition from pediatric to adult-based dental homes was not correlated with the readiness to transition
3. Multiple emotional and logistical barriers affected caregiver desire to transition their adolescent to an adult-centered dental home

## Figures and Tables

**Table 1:** Demographic information.

Variable	N (%)
Age of patient	Mean: 17 median: 15
12–15	17 (63)
16–19	8 (29.6)
20–23	1 (3.7)
>23	1 (3.7)
Type of residence	
Home with family	23 (85.2)
Inpatient residence facility	2 (7.4)
Other	2 (7.4)

**Table 2:** Fisher's exact test of the association between awareness of the need to transition and readiness for the patient to transition to adult-based care.

	Readiness for transition		Total	
Awareness of the need to transition	Ready	Not ready		
Unaware	14	3	17	
Aware	5	5	10	
	19	8	Two-sided Pr ≤ P	0.10

## Data Availability

The data used to support the findings of this study are available from the corresponding author upon request.
